# Effectiveness of conditional cash transfers, subsidized child care and life skills training on adolescent mothers’ schooling, sexual and reproductive health, and mental health outcomes in Burkina Faso and Malawi: the PROMOTE Project pilot randomized controlled trial protocol

**DOI:** 10.1186/s12978-023-01706-9

**Published:** 2023-11-09

**Authors:** Caroline W. Kabiru, Alister Munthali, Nathalie Sawadogo, Anthony Idowu Ajayi, Catherine Asego, Patrick G. Ilboudo, Anne M. Khisa, Grace Kimemia, Beatrice Maina, Jane Mangwana, Michelle Mbuthia, Ramatou Ouedraogo, Chrissie Thakwalakwa, David Wanambwa, Alexandra Tapsoba, Witness Olex Tapani Alfonso

**Affiliations:** 1 African Population and Health Research Center (APHRC), APHRC Campus, Manga Close, Off Kirawa Road, Nairobi, Kenya; 2 Centre for Social Research (CSR), University of Malawi, Zomba, Malawi; 3 Institut Supérieur des Sciences de la Population (ISSP), Université Joseph Ki-Zerbo, Ouagadougou, Burkina Faso

**Keywords:** Adolescent mothers, Schooling, Randomized controlled trial, Interventions, Malawi, Burkina Faso

## Abstract

**Introduction:**

Girls’ and women’s health as well as social and economic wellbeing are often negatively impacted by early childbearing. In many parts of Africa, adolescent girls who get pregnant often drop out of school, resulting in widening gender inequalities in schooling and economic participation. Few interventions have focused on education and economic empowerment of adolescent mothers in the region. We aim to conduct a pilot randomized controlled trial in Blantyre (Malawi) and Ouagadougou (Burkina Faso) to examine the acceptability and feasibility of three interventions in improving educational and health outcomes among adolescent mothers and to estimate the effect and cost-effectiveness of the three interventions in facilitating (re)entry into school or vocational training. We will also test the effect of the interventions on their sexual and reproductive health (SRH) and mental health.

**Interventions:**

The three interventions we will assess are: a cash transfer conditioned on (re)enrolment into school or vocational training, subsidized childcare, and life skills training offered through adolescent mothers’ clubs. The life skills training will cover nurturing childcare, SRH, mental health, and financial literacy. Community health workers will facilitate the clubs. Each intervention will be implemented for 12 months.

**Methods:**

We will conduct a baseline survey among adolescent mothers aged 10–19 years (N = 270, per site) enrolled following a household listing in select enumeration areas in each site. Adolescent mothers will be interviewed using a structured survey adapted from a previous survey on the lived experiences of pregnant and parenting adolescents in the two sites. Following the baseline survey, adolescent mothers will be individually randomly assigned to one of three study arms: arm one (adolescent mothers’ clubs only); arm two (adolescent mothers’ clubs + subsidized childcare), and arm three (adolescent mothers’ clubs + subsidized childcare + cash transfer). At endline, we will re-administer the structured survey and assess the average treatment effect across the three groups following intent-to-treat (ITT) analysis, comparing school or vocational training attendance during the intervention period. We will also compare baseline and endline measures of SRH and mental health outcomes. Between the baseline and endline survey, we will conduct a process evaluation to examine the acceptability and feasibility of the interventions and to track the implementation of the interventions.

**Discussion:**

Our research will generate evidence that provides insights on interventions that can enable adolescent mothers to continue their education, as well as improve their SRH and mental health. We aim to maximize the translation of the evidence into policy and action through sustained engagement from inception with key stakeholders and decision makers and strategic communication of research findings.

*Trial registration number* AEARCTR-0009115, May 15, 2022.

## Background

Early childbearing in sub-Saharan Africa is associated with negative impacts on girls’ physical and mental health, and socio-economic wellbeing including school dropout [1–3]. Research has shown that many adolescent mothers would like to return to school but lack the needed support to pursue their educational goals [2, 4]. While some countries in the region have policies that ensure that pregnant girls are supported to continue with learning during pregnancy and return to school after they have delivered, their implementation is lacking. Instead, girls who get pregnant while at school face stigma, abuse, violence, or rejection and often receive little or no support from their families, school administrators and the community [4]. These issues present serious challenges for pregnant girls and adolescent mothers hoping to continue their education [5]. Without developing effective approaches to ensure the re-entry into school for the millions of girls dropping out of schools each year because of unintended pregnancy, sub-Saharan African countries will not achieve gender equality in schooling.

Adolescent mothers are also at risk for rapid repeat pregnancies (defined as a second pregnancy within 24 months of the first pregnancy), if not provided with adequate care and support [6]. Such closely spaced births further exacerbate their risk of adverse pregnancy outcomes and negative consequences [7, 8]. Research has shown that adolescent mothers in sub-Saharan Africa have the highest prevalence of short birth spacing [9]. In a recent analysis, the overall prevalence of contraceptive use among adolescents in the region was 21%, ranging from 70% in South Africa to only 5% in Chad [10]. In one study conducted in Kenya, women who gave birth between the ages of 15 and 24 years were more likely to discontinue postpartum contraceptive use than women who gave birth at older ages [11]. Investing in their postpartum contraceptive use has several health and socioeconomic benefits, including allowing adolescent mothers to pursue education and vocational skills development and protect their physical and mental health. Such investment would have twin benefits including preventing repeat pregnancy and HIV and other sexually transmitted infections (STIs).

Despite the negative consequences of adolescent childbearing for girls’ education, health and social wellbeing, and gender equality, few interventions have focused on education and economic empowerment of parenting adolescents in Africa. Improving health and social outcomes for pregnant and parenting adolescents will require interventions that challenge the gendered social norms and hierarchies that perpetually limit parenting girls’ education, financial opportunities and economic security. Promising multilevel interventions for girls’ empowerment like the Adolescent Girls Initiative—Kenya (AGI-K), have been shown to lead to violence reduction, primary school completion, increased sexual and reproductive health (SRH) knowledge, financial literacy and savings, and positive gender norms [12]. However, it is unclear if, at all, such interventions can increase adolescent mothers’ re-entry into school and vocational training, and improve SRH and mental health outcomes among them.

To address this gap, we aim to implement a pilot randomized controlled trial (RCT) in Burkina Faso and Malawi to address the social exclusion of adolescent mothers and foster their re-entry into school or vocational training while enhancing their life skills. We chose Burkina Faso and Malawi because both countries face a huge burden of adolescent childbearing but have contrasting policy contexts on school re-entry. While girls’ education remains a key priority of decision-makers in Burkina Faso [13], there is no clear policy or guideline for supporting pregnant girls in schools. In Malawi, the Readmission Policy for Primary and Secondary Schools allows school-aged mothers to resume school after giving birth, reversing a previous policy that banned them from re-enrolling [14, 15]. Under the current policy, after delivery, adolescent mothers can request for readmission from both the Ministry of Education and the school [14]. However, the extent to which this policy is effective in facilitating school re-entry of parenting adolescents is unknown.

The three interventions we will assess are: (1) a cash transfer conditioned on (re)enrolment into school or vocational training and continued attendance; (2) subsidized child care; and (3) adolescent mothers’ clubs where adolescent mothers will receive life skills training covering nurturing childcare, SRH, and financial literacy. The choice of these interventions is informed by previous research highlighting financial constraints and lack of childcare as major barriers to school re-entry for parenting adolescents [3, 4]. These interventions also build on existing initiatives that provide a platform for scaling up should our research demonstrate their effectiveness. For example, the Government of Malawi runs the Social Cash Transfer Programme (SCTP), which provides ultra-poor and labor-constrained households an unconditional monthly transfer of about 7000 Malawian Kwacha (~ US$ 8.7) [16]. Currently, adolescent mothers are not targeted in the SCTP. The SCTP provides a framework for implementing cash transfers to parenting adolescents.

The specific objectives are to: (1) test the feasibility and acceptability of the interventions in the study contexts; (2) determine which combination(s) of interventions (cash transfer, subsidized child care, and adolescent mothers clubs) lead to the greatest increase in adolescent mothers’ re-entry into school or vocational training (primary outcome); (3) assess the effects of different combinations of the interventions on SRH and mental health outcomes among adolescent mothers (secondary outcomes); and (4) assess the cost-effectiveness of these interventions in increasing adolescent mothers’ re-entry into school or vocational training.

We hypothesize that these interventions will enhance adolescent mothers’ social, economic, health and education assets thereby allowing them to (re)enroll into school or vocational training. We also hypothesize that building these assets will lead to improved SRH and mental health outcomes for these mothers. Additionally, we hypothesize that combining the conditional cash transfer, subsidized child care and adolescent mothers’ club interventions is more effective in increasing re-entry into school and vocational training than adolescent mothers’ clubs only or combined adolescent mothers’ clubs and subsidized child care interventions.

## Theoretical framework

Our research is guided by the ecological framework, which underscores the importance of an enabling environment for adolescents’ health and development. Barriers that hinder pregnant and parenting adolescent girls’ education and economic empowerment operate at multiple levels, including at individual, household and community levels. Interventions that address these multiple levels of barriers are more likely to be effective than those that focus on a single level [17].

At the individual level, it is important to focus on interventions that empower parenting adolescents by building their social, economic, health and education assets. In this respect, our proposed interventions seek to empower adolescent mothers by providing them with cash transfers conditioned on re (enrolment) into school or vocational training and continued attendance. Adolescent mothers will also receive life skills training on SRH, nurturing care for children, and financial literacy. The training will be provided through adolescent mothers’ clubs, which are modeled on the Population Council’s Safe Spaces groups [18], where adolescent mothers will meet in groups under the guidance of a community health worker. The community health workers (CHWs) will also support adolescent mothers in accessing contraceptives and other SRH services through referrals to adolescent-friendly SRH providers. Further, the clubs will help in creating social solidarity and serve as a social support network to empower adolescent mothers to collectively address the issues they face in their daily lives including social stigma associated with adolescent pregnancy. In light of research showing that lack of child care is a significant barrier to school re-entry for parenting adolescents, our intervention will also include vouchers for subsidized child care in registered childcare facilities/crèches.

Given the potential for contamination, we will not test a community wide intervention. However, we will conduct community dialogues aimed at engaging a variety of community members including community and religious leaders, health workers, social workers, teachers, and parents. The goal of these community dialogues will be to identify the root causes of adolescent pregnancies, including gender norms; to discuss possible ways to support parenting adolescents, including addressing stigma against pregnant and parenting adolescents; and to develop a sense of ownership in collectively supporting these adolescents.

**Fig. 1:**
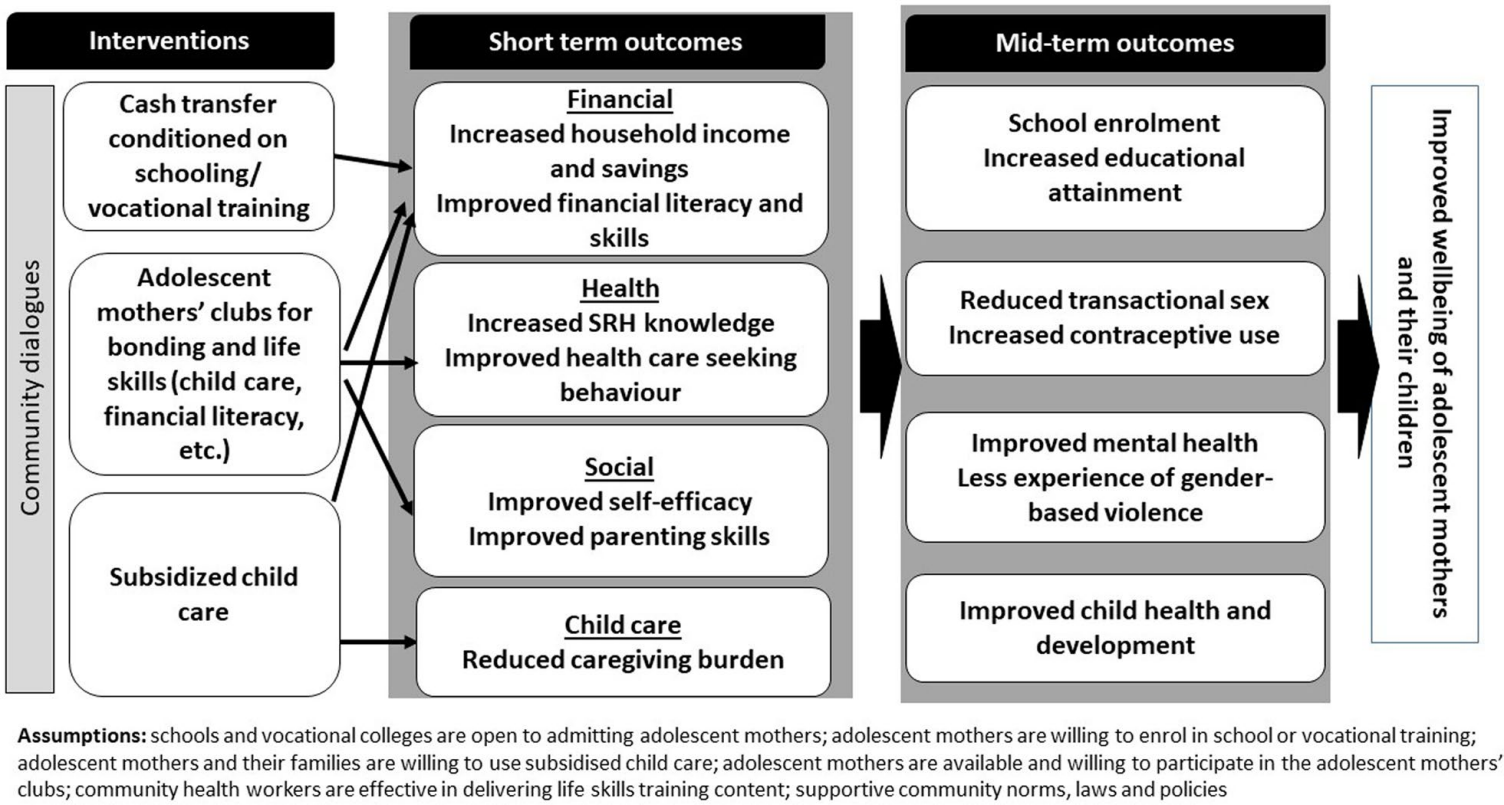
PROMOTE project theory of change

While our interventions do not address societal level factors, our research uptake activities will focus on promoting laws (including community by laws) and policies that support parenting adolescents to realize their rights, including their rights to education and comprehensive SRH interventions. Figure 1 summarizes our theory of change.

## Methods

### Study design

**Fig. 2:**
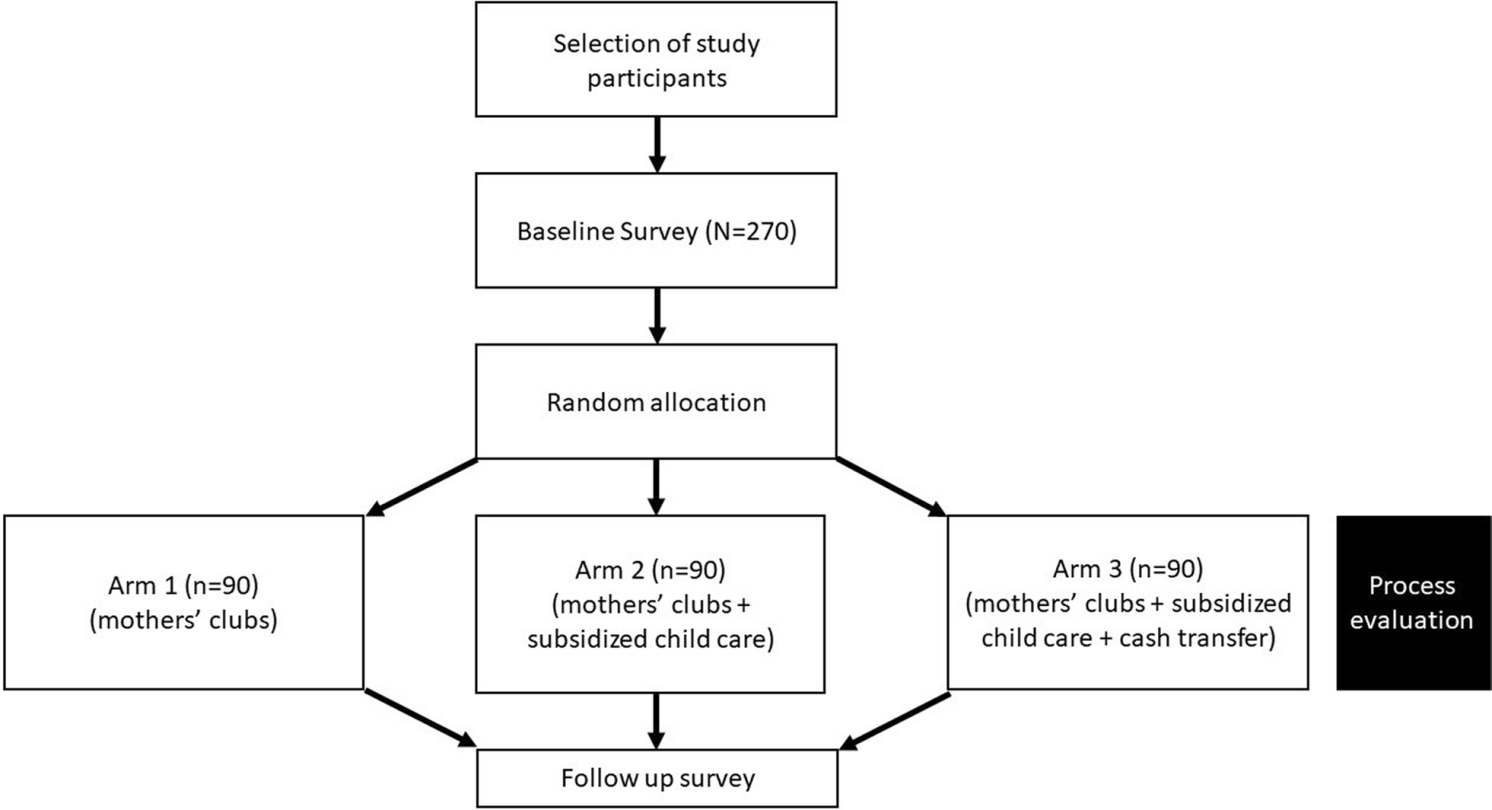
Pilot randomized controlled trial design for the PROMOTE Project

We will conduct a pilot RCT designed to estimate the effect of a conditional cash transfer and subsidized child care on adolescent mothers’ re-entry into school or vocation training. We also seek to examine the effects of the interventions on SRH outcomes and mental health as secondary outcomes, and to determine the most cost-effective interventions to increase adolescent mothers’ re-entry into school or vocational training. We will randomly assign adolescent mothers to one of three study arms. Adolescent mothers in all the arms will receive life skills training through adolescent mothers’ clubs. Adolescent mothers in arm two will also receive subsidized child care, while those in arm three will receive all three interventions. Comparing arm one (mothers’ clubs only) and arm two (mothers’ clubs + subsidized child care) will allow us to test the additional benefit of the subsidized child care. Comparing arms two and three (mothers’ clubs + subsidized child care + cash transfer) will also allow us to test the additional benefit of the cash transfer. Comparing arms one and three will allow us to test the combined benefit of the subsidized child care and cash transfers. The RCT design is summarized in Fig. 2.

### Study sites

The study will be conducted in urban settings in Ouagadougou (Burkina Faso) and Blantyre District (Malawi). We selected these settings given previous research showing low school enrollment among pregnant and parenting adolescents. In a survey conducted in Ouagadougou, Burkina Faso, 82% of pregnant and parenting adolescents were out of school, with 23% reporting that they dropped out of school because of pregnancy [19]. Among those out of school in Burkina Faso, 39% wanted to return to school, while 79% wanted to have an income generating activity. Girls reported the lack of childcare support, stigma, and inadequate support as some of the barriers to school re-entry.

A similar survey conducted in Blantyre District in Malawi found that poverty, sexual violence, inequitable gender norms, and lack of access to accurate contraceptive information and services make girls vulnerable to early and unintended pregnancy [4]. Most girls get pregnant outside of marriage and consequently face social exclusion from their peers, parents and communities. While most rely on their parents to provide for them and their babies, some depend on their partners. However, most face dire financial situations, sometimes lacking food and other basic needs. Approximately 94% of adolescents surveyed were out of school, and about half of them dropped out because of pregnancy. However, 76% of them wanted to return to school if provided with financial and childcare support, while 93% wanted to learn vocational skills [4].

### Interventions

Each intervention will be implemented for 12 months. Below, we describe each intervention:

#### Adolescent mothers’ clubs

The adolescent mothers’ clubs will be held every two weeks for 12 months in a variety of locations in the community considered by adolescent mothers to be safe and appropriate for them to meet. During the club meetings, adolescent mothers will receive life skills training facilitated by trained CHWs. The club will create a safe space where adolescent mothers can interact to discuss their common problems, learn about child care, mental health, HIV, SRH (including harmful gender norms that raise the risk of experiencing and tolerating sexual and gender-based violence), conflict resolution, hygiene, and financial literacy (e.g., making a savings plan, spending priorities, and where to save).

Because the burden of poor mental health is significant among adolescent mothers [20], the club will also offer a safe space where these adolescent mothers can receive psychosocial support from CHWs and peers. The collectivism the club creates also has the potential for strengthening their self-esteem, a resource they need to address the stigma associated with adolescent childbearing. Given their role as a bridge between the community and the health sector, we also expect that the CHWs will also facilitate adolescent mothers’ access to SRH and child health services. CHWs will also refer any adolescent mothers needing additional psychosocial support to child protection workers, who work at community level.

The CHWs will follow a structured curriculum that has been adapted from the Population Council’s AGI-K Safe Spaces curriculum. [21]; the Mphatlalatsane (Early Morning Star) Health and Nutrition Programme Manual [22]; the Maternal and Child Health Handbook [23] and the Parenting for Lifelong Health Programme for Young Children (Facilitator manual) [24]. The CHWs will undergo a 5-day training workshop on the curriculum. The workshop will be facilitated by the research team and will adopt a participatory approach that will also serve to demonstrate the participatory methods to be used by CHWs during the adolescent mothers’ clubs.

Each adolescent mothers’ club will include about 15–25 adolescent mothers living within the same locality. The sessions will be approximately 2 h long. CHWs will monitor and record attendance and will follow up adolescent mothers who miss sessions.

#### Subsidized child care

Vouchers have been effectively used for childcare in Kenya [25]. Adolescent mothers in arms two and three will receive vouchers for subsidized childcare in selected childcare centers in the two study sites. Adolescent mothers who receive the vouchers will be entitled to 12 months of subsidized childcare. In Malawi, we will work with community-based childcare centers (CBCCs), which are managed by community members and overseen by the Ministry of Gender, Community Development and Social Welfare. These centers depend on contributions from the community and parents are expected to contribute a nominal amount towards the running of these centers [26]. In Burkina Faso, we will work with a mix of public and private preschools as well as community-based childcare centers. In the inception period, we will map out childcare centers and conduct brief interviews with the childcare center managers/teachers to understand the level of financial support needed. The information obtained will help us identify specific centers where adolescent mothers can send their children, as well as help us establish a reasonable voucher amount. Adolescent mothers who are eligible to receive vouchers will receive 12 vouchers. They will hand over a voucher to the respective childcare center at the beginning of every month. In each study setting, field staff will collect the vouchers from each childcare center to monitor the intervention, as well as to determine the exact amount to be transferred to each childcare center.

#### Conditional cash transfers

The cash transfer will be conditioned on (re)enrolment in primary or secondary school (junior and senior high school in Burkina Faso) or vocational training programs. A Cochrane systematic review [27] showed that cash transfer programs that are conditional and that incorporate systems to monitor compliance and that penalize non-compliance have greater effects on enrolment. Transfers will be made for every term during the intervention period that the girl is enrolled and remains enrolled. During the intervention period, field staff will visit schools at regular intervals to confirm adolescent mothers’ continued enrolment.

Adolescent mothers in arm three will receive a cash transfer every three months. Each transfer will be approximately US$ 30 (US$ 10 per month) and will be transferred through mobile money platforms, which are common in Burkina Faso and Malawi. Cash transfers to mobile phone-based money transfer services have been successfully used in similar settings [28].

### Outcomes

#### Primary outcome

The primary outcome of this study is (re)entry or (re)enrolment in school or vocational training. At baseline, all participants will respond to a question on current school and vocational training enrolment and their intention to return to school or vocational training. At endline, participants will be asked whether they have attended school or vocation training at any time after the baseline. The primary outcome will be a binary variable coded 1 “Yes” or 0 “No” indicating whether or not the participant was enrolled in school or vocation training during the 12-month long intervention period.

#### Secondary outcomes

We will assess five secondary outcomes related to SRH and mental health. Table 1 summarizes the secondary outcomes to be assessed. We will compare baseline and endline measures of these outcomes.

**Table 1:** Secondary outcomes to be assessed

Domain	Secondary outcome	Measurement
SRH	SRH knowledge	To be measured using a series of questions assessing adolescent mother’s knowledge of HIV and its transmission, the menstrual cycle, and contraceptive methods
	Current contraceptive use	To be assessed using two questions assessing current use of a contraceptive method to delay or avoid getting pregnant and the type of method used
	Transactional sex	To be measured using a series of questions on self-reported exchange of sex for money, girl, rent, food, school fees, phone/airtime, clothes/shoe/beauty products, sanitary pads, and security
	Experience of sexual and gender-based violence	To be assessed using a series of questions adapted from the *WHO multi-country study on women’s health and domestic violence against women* [29]
Mental health	Mental health	To be measured using the Patient Health Questionnaire-9 (PHQ-9) tool, which has been used in other settings in sub-Saharan Africa [30, 31], to identify adolescent mothers with depression symptoms

### Covariates

The following covariates will be considered in comparing intervention effectiveness outcomes between the intervention arms: age, socio-economic status, family structure, family support, marital status, living arrangements, employment status, and neighborhood safety nets and assets.

### Sample size and sample selection

Aside from using the pilot study to assess the feasibility and implementation of the intervention, we also aim to ensure that the pilot study provides us with preliminary data to accurately estimate the sample size for a fully-fledged intervention study in the future. That is, we expect the pilot data to enable accurate estimation for the within-group variance, intraclass correlation, and estimate of true effect size for a larger study. Thus, we approach the sample size estimation through a formal but naïve approach assuming there are two groups to compare (instead of three, and not accounting for multiplicity). We consider (re)enrolment in school or vocational training as the primary outcome. The sample size is estimated using the following parameters: in the lived experiences survey implemented in Malawi, the proportion of pregnant or parenting adolescents who were currently out of school was 94%. In a study conducted by the Population Council in Kenya, the proportion of adolescent mothers re-entering school following an intervention comprising policy dialogue, values clarification, and advocacy ranged from 10 to 16% [32]. Given that our proposed intervention addresses the key barriers to school re-entry identified in the Population Council study, we expect a higher rate of re-entry. For this study, we shall assume that the intervention, at the minimum, will achieve a 15% absolute reduction in the proportion of adolescents not in school. The minimum total required sample size for detecting this difference between two groups for a two-sided level of significance of 10%, and statistical power of 80%, can be computed as:

$$n=\frac{{[{Z}_{1-\frac{\alpha }{2}}\sqrt{2\overline{pq} }+{Z}_{1-\beta }\sqrt{{p}_{1}\left(1-{p}_{1}\right)+{p}_{2}\left(1-{p}_{2}\right)}]}^{2}}{{({p}_{1}-{p}_{2})}^{2}}$$

${p}_{1}$ is the proportion of pregnant or parenting adolescents who will be out of school in the intervention groups; ${p}_{2}$ is the proportion for the arm receiving only the mothers club.; $n$ is minimum sample size per arm;$\overline{p }=\frac{{p}_{1}+{p}_{2}}{2}$, and$\overline{q }=1-\overline{p }$. Assuming a refusal or non-consent or attrition rate of 15% and a design effect of 1.1 due to possible clustering of adolescents from the same enumeration area (EA), the required sample size for detecting a difference between the control arm (group receiving only mother club membership) and one intervention arm is 90 adolescents per arm. In this pilot study with three arms, a total sample size of 270 adolescent mothers is needed.

We will select adolescent mothers using multi-stage sampling. In the first stage, we will select EAs from the primary sampling frames developed by the National Statistical Office for the 2018 Population and Housing Census in Malawi, and by the Burkina Faso’s Institut National de la Statistique et de la Démographie in 2006 and updated in 2017. In selecting the enumeration areas, we will consider the availability of childcare centers and venues to host the adolescent mothers’ clubs. In the second stage, we will conduct a household listing in the selected EAs to identify households with eligible participants. In households with eligible adolescents, we will randomly select one using a Kish grid. The adolescents identified will be invited to participate in the baseline and endline surveys and the intervention.

### Eligibility criteria

Eligible participants will comprise adolescent mothers aged 10–19 years who:

- Have at least one biological child aged 1–3 years.
- Can speak and understand the main language used in the mothers’ clubs in the study context.
- Have been resident in the study site for at least 1 year.
- Consent/assent to being in the study (and, for minors, whose parents/guardians provide consent).

Adolescent mothers who are living with disability will be eligible to participate as long as they meet the eligibility criteria.

### Exclusion criteria

- Currently pregnant.
- Unable to participate in the adolescent mothers’ clubs.

### Randomization and data collection

In each site, adolescents mothers will be interviewed at baseline and endline using a structured questionnaire drawn from the ‘*Understanding the lived experiences of pregnant and parenting adolescents in Burkina Faso and Malawi*’ study [4, 19]. The questionnaire includes questions on adolescent mothers’ sociodemographic and background characteristics, including their schooling and desire to return to school; family characteristics; social capital and networks; self-reported physical and mental health; marriage and sexual behavior; SRH knowledge and contraceptive behavior; pregnancies and births; HIV/AIDS and other sexually transmitted infections; gender-based violence and adverse childhood experiences; aspirations, concerns and perceived life chances; and self-efficacy.

At the end of the baseline survey, adolescent mothers will be individually randomly assigned to one of the three intervention groups using computer generated random numbers. CHWs facilitating the adolescent mothers’ clubs will be blinded to the adolescent mothers’ study arm.

### Process evaluation

Between the baseline and follow-up survey, we will conduct a process evaluation. The process evaluation will seek to: understand adolescent mothers’ and other key stakeholders’ perceptions of the interventions including barriers and facilitators in taking up the interventions assess the feasibility of delivering the three interventions in the study contexts; and assess the uptake of the three interventions.

#### Targeted population and approach

The process evaluation will use quantitative and qualitative approaches. The qualitative approach will include observations of the adolescent mothers’ clubs, repeated in-depth interviews (IDIs) with 21 of the adolescent mothers to understand their experiences with the interventions as well as the barriers and facilitators to (re)enrolling in school or vocational training and in accessing childcare services. In addition, key informant interviews will also be conducted with heads of schools/vocational training centers where these adolescent mothers are enrolled, managers of the child care centers where the children of the adolescent mothers are enrolled and parents/partners of the adolescent mothers participating in this program. In addition, we will also observe some activities of the intervention, including the mothers’ clubs and day-care visits to capture the dynamics in such spaces, the information provided to adolescent mothers and how they relate to the information, while documenting their effective presence and that of their child.

For the IDIs, we will purposively select 21 adolescent mothers. These adolescents will be selected from the three intervention arms: seven per arm. To capture a diversity of experiences, we will aim to have a sample comprising adolescent mothers of different ages, religion, schooling status, occupation, living arrangements and marital status, as well as from different areas of residence. Upon selection, we will conduct an initial IDI using a semi structured guide to document their living conditions as parenting adolescents, including challenges experienced, relations with their relatives and partners, reasons for dropping out of school, stigma, adolescent mothers’ knowledge on parenting and the type of support they receive from relatives, reproductive and mental health issues, among other issues. We will then conduct follow up interviews every 3 months to document changes in their living conditions, including their experiences with the interventions as well as the barriers and facilitators to (re) enrolling and remaining in school or vocation training and in accessing childcare and other services. The initial interview will be used as the basis for questions for the follow-up interviews. The interviews will take place at home or a venue suitable for the adolescent mothers.

We will also interview key actors involved in adolescent mothers’ lives such as head teachers in schools where these adolescent mothers are enrolled, managers of the childcare center where the children of the adolescent mothers are enrolled, as well as parents and partners of the adolescent mothers participating in this program. These interviews will aim at capturing their perspectives about the program, its impact on their practices and behaviors towards adolescent mothers.

In addition to individual level observations, we will also follow up the adolescent mothers as they engage with some activities of the intervention, including the mothers’ clubs and day-care visits to capture the dynamics in such spaces, the information provided to adolescent mothers and how they relate to the information, while documenting their effective presence and that of their child. The research team will document observations using field notes.

The qualitative data will be complemented by administrative data on the costs of implementing the interventions, attendance in the adolescent mothers’ clubs, use of childcare centers, as well as adolescent mothers’ enrolment in school and vocational training. This data will be gathered from the financial reports, as well as the day-care and school registries.

### Cost-effectiveness evaluation

The cost-effectiveness evaluations will examine the value for money of the three interventions in promoting adolescent mothers’ (re)entry and enrolment into school or vocational training, and document the additional value of the proposed interventions on SRH and mental health outcomes. The cost-effectiveness analysis will be carried out from the government-funded program perspective. The costing assessment will focus on the total costs that would be incurred by the government in delivering the integrated interventions. More specifically, we will assess the annual incremental costs of the delivery of each intervention including start-up and implementation costs.

The start-up costs will capture all the costs incurred for the preparation and introduction of the interventions while the implementation costs will consist of the costs incurred for the implementation of the intervention activities. These include incurred costs for the identification and hiring of program staff, identification of childcare centers to be involved in the intervention, development of the system for administering and monitoring the childcare voucher system, setting up of the cash transfer system, inception meetings including CHWs, initial training, cost associated with mentoring CHWs as they deliver the interventions, costs for sensitization and awareness-raising activities, supervision/monitoring during start-up as well as cost associated with overall coordinating activities and capital items acquired in the start-up. The start-up costs will be annualized accordingly.

The implementation costs will encompass all relevant costs such as the costs for mentoring and supervision, replenishment of capital items, mothers’ club operation costs, printing and materials, vouchers paid to childcare centers, amounts transferred in cash to adolescent mothers, and time invested by CHWs in facilitating the adolescent mothers’ clubs.

#### Data sources

To estimate these costs, we will use both primary and secondary data. Primary data will be obtained by collecting self-reported estimates of time use data from CHWs delivering adolescent mothers’ clubs. Secondary data will be collected by capturing the costs associated with launching and rolling out the interventions. The former costs include salaries and stipends (for CHWs), mobilization and sensitization, training, material, transport (including for mentoring and monitoring purposes), mentorship and supervision, and per diems, and will be determined by analyzing implementers’ financial documents as well as budgets.

### Data processing and analysis

Outcomes will be measured pre-randomization (at baseline) and at the end of the 12-month intervention period (endline). Quantitative data will be collected on Android-based tablets programmed using SurveyCTO. The data will be synchronized to a password protected server. The data will then be transferred to Stata for further cleaning and analyses.

At the endline, we will estimate the average treatment effect across the three groups using intent-to-treat (ITT) analysis, comparing school/vocational training enrolment at baseline and endline. Primary analyses will be unadjusted. We will also compute adjusted effects (controlling for covariates) to account for differential attrition or any differences at baseline. Drawing on financial records detailing the cost of the interventions (cash transfers, childcare vouchers, and cost of running the mothers’ clubs), we will conduct cost-effectiveness analyses of the interventions. The cost per (re)enrolled adolescent mother will be the primary measure of the cost-effectiveness of the interventions. This costing data per adolescent mother will be further combined with secondary effectiveness data to calculate additional cost-effectiveness estimates of the interventions. Secondary effectiveness data will be measured through improvements in mental health of adolescent mothers and improved knowledge and uptake of SRH practices.

All recorded qualitative interviews undertaken for the process evaluation will be translated and transcribed into English by bilingual transcribers. The interviews from Burkina Faso will be initially transcribed directly in French from local languages. The transcripts and field notes will then be translated into English in order to obtain bilingual transcripts. To ensure the accuracy of transcription, we will compare the transcripts of a selected number of interviews with the original recordings. The transcripts and field notes will be imported into NVivo (or other software) for data coding. Two researchers will independently code the data. We will develop a codebook using both inductive and deductive approaches, that is, using the study objectives as the starting point but also expanding as we read and re-read the transcripts. Before coding the transcripts, transcripts will first be read for familiarity and then followed by a more reflexive and critical reading. At this stage, data will be grouped into issues that are directly related to the study objectives. Once the coding is completed, we will compare the codes generated by the two researchers and group the final codes to themes. Using thematic analysis and the field notes, we will ensure that all relevant information is coded and grouped into themes. We will explore each theme to create subthemes. Our themes will be discussed with both research teams to ensure they accurately capture respondents’ narratives. An experienced qualitative researcher will analyze a random sample of our data and field notes to ensure the credibility of our analysis. We will ensure that views, both supportive of or against our thesis, are accurately reflected in our analysis. Verbatim quotes will be used to support emerging themes from our qualitative findings.

### Potential study limitations

While we will adopt a rigorous study design, there are several potential limitations of the study. First, although the selection of the interventions was informed by findings from surveys of pregnant and parenting adolescent girls in Burkina Faso and Malawi and dialogues with community members and other stakeholders, including adolescent mothers, these interventions may be perceived to have been selected in a top-down rather than participatory approach. Second, the one-year duration of the RCT may be too short to observe any effects particularly if there are restrictions on when adolescent mothers can (re)enroll in school or vocational training programs. Finally, there may be differential attrition by study arm between baseline and endline that may limit our ability to draw conclusive evidence on the effects of the interventions.

## Discussion

We expect that our research will generate rigorous evidence that can inform policies and programs aimed at promoting the wellbeing of pregnant and parenting adolescents. Our research builds on existing studies on the lived experiences of pregnant and parenting adolescents that were informed in part by inputs from various stakeholders in both countries. In addition, the interventions are informed by emerging evidence from these studies that underscore financial and childcare constraints as critical barriers to school re-entry for these adolescents. We therefore expect that our proposed interventions will respond to needs highlighted by pregnant and parenting adolescents themselves—majority of whom are out of school but express a desire to return to school and the wider community.

Community health workers play an important role in enhancing communities’ access to preventative health services including health promotion and education services [33]. In this study, we propose to work with CHWs to deliver life skills education to adolescent mothers through adolescent mothers’ clubs. Given their role as a bridge between the community and the health sector, CHWs may also facilitate adolescent mothers’ access to SRH, child health, and psychosocial services. This is particularly critical given the significant challenges that adolescent mothers face in accessing health services [34]. Through this project, we therefore expect to demonstrate the role that this cadre of health workers plays in enhancing adolescent mothers’ trust in the health system and, consequently, improving their health care seeking behavior.

## Data Availability

The research will generate anonymized, individual level qualitative and quantitative data capturing, among other indicators, sociodemographic characteristics, SRH, schooling and employment data from adolescent mothers. These data can be mined by other researchers seeking to answer a variety of public health and sociological questions relevant to adolescent and youth populations. Importantly, some of the data will be longitudinal and can be used to make causal inferences. De-identified data will be made publicly available for further analyses following a 2-year embargo after the finalization of the study.

## Abbreviations

AGI-K: Adolescent Girls Initiative-Kenya
CHW: Community health worker
EA: Enumeration area
IDI: In-depth interview
ITT: Intent-to-treat
PHQ-9: Patient Health Questionnaire-9
RCT: Randomized controlled trial
SRH: Sexual and reproductive health
SCTP: Social Cash Transfer Programme (Malawi)
STI: Sexually transmitted infection
UNIMAREC: University of Malawi Research Ethics Committee
